# Treatment of Febrile illness with artemisinin combination therapy: prevalence and predictors in five African household surveys

**DOI:** 10.1186/s40545-014-0024-0

**Published:** 2015-01-31

**Authors:** Catherine E Vialle-Valentin, Robert F LeCates, Fang Zhang, Dennis Ross-Degnan

**Affiliations:** Department of Population, Medical Harvard School and Harvard Pilgrim Health Care Institute, 133 Brookline Avenue, 6th Floor, Boston, MA 02215 USA

**Keywords:** Artemisinin combination therapy, Household surveys, Appropriate use of medicines, Africa

## Abstract

**Objectives:**

To evaluate the determinants of compliance with national policies recommending Artemisinin Combination Therapy (ACT) for the treatment of uncomplicated malaria in the community.

**Methods:**

We used data from Gambia, Ghana, Kenya, Nigeria, and Uganda national household surveys that were conducted with a standardized World Health Organization (WHO) methodology to measure access to and use of medicines. We analyzed all episodes of acute fever reported in the five surveys. We used logistic regression models accounting for the clustered design of the surveys to identify determinants of seeking care in *public* healthcare facilities, of being treated with antimalarials, and of receiving ACT.

**Results:**

Overall, 92% of individuals with a febrile episode sought care outside the home, 96% received medicines, 67% were treated with antimalarials, and 16% received ACT. The choice of provider was influenced by perceptions about medicines availability and affordability. In addition, seeking care in a *public* healthcare facility was the single most important predictor of treatment with ACT [odds ratio (OR): 4.64, 95% confidence intervals (CI): 2.98–7.22, P < 0.001]. Children under 5 years old were more likely than adults to be treated with antimalarials [OR: 1.28, CI: 0.91–1.79, not significant (NS)] but less likely to receive ACT (OR: 0.80, CI: 0.57–1.13, NS).

**Conclusions:**

Our results confirm the high prevalence of presumptive antimalarial treatment for acute fever, especially in public healthcare facilities where poor people seek care. They show that perceptions about access to medicines shape behaviors by directing patients and caregivers to sources of care where they believe medicines are accessible. The success of national policies recommending ACT for the treatment of uncomplicated malaria depends not only on restricting ACT to confirmed malaria cases, but also on ensuring that ACT is available and affordable for those who need it.

## Introduction

Artemisinin-Combination Therapy (ACT) is considered the most effective pharmaceutical intervention to control malaria, in conjunction with long-lasting insecticidal nets (LLIN), indoor residual spraying (IRS) and intermittent presumptive treatment (IPT) with Sulfadoxine/Pyrimethamine (SP) during pregnancy [1-5]. In recent years, national policies have endorsed first-line treatment with ACT for uncomplicated malaria in countries at high risk of malaria. Implementation of such policies presents considerable challenges on the supply side to ensure adequate funding through international subsidies, sufficient manufacturing of co-blistered combinations in line with internationally recommended standards, and uninterrupted distribution [6,7]. On the demand side, deep-rooted treatment behaviors and high ACT retail prices have slowed the adoption of the policy by healthcare providers and patients alike [8,9].

*ACTWatch* household surveys conducted at the start of the Affordable Medicines Facility-malaria (AMFm) initiative found that less than half of children under 5 years old with fever received ACT [10-13]. Little evidence exists about the attitudes and perceptions of patients and caregivers regarding national recommendations to switch from well-established practices to new treatment paradigms. Yet, a better understanding of the factors slowing the adoption of ACT is needed [14]. The objectives of this study were to describe the early compliance with national policies recommending ACT as first-line therapy for suspected malaria and to identify predictors of ACT use in communities at high risk of malaria. To investigate the extent of ACT utilization by households and the determinants of ACT use, we analyzed existing data from household surveys that were conducted with a standard methodology developed by the World Health Organization (WHO) to measure medicines access and use in low- and middle-income countries.

## Methods

### Survey methodology, data collection and management

The WHO methodology to measure medicines access and use with standardized rapid cluster sample household surveys has been previously described [15]. This study includes five surveys in countries at high risk of malaria that were conducted with the original WHO methodology (The Gambia, Nigeria) or the revised Medicines Transparency Alliance (MeTA) methodology (Ghana, Kenya, and Uganda). Data collection took place in August 2007 (Nigeria), October 2007 (The Gambia), May-June 2008 (Ghana), July-August 2008 (Uganda) and September-October 2008 (Kenya). The open source software EpiData Entry v.2.0 (The EpiData Association, Odense, Denmark) was used for data entry. All medicines names were entered as collected and their equivalent generic nomenclature was coded with a Microsoft Excel^tm^ tool based on the WHO 15^th^ Model Essential Medicines List [16]. All medicines coded 6.531 “antimalarial” are included in this analysis.

### Analysis

We received anonymized country survey datasets from local teams after obtaining Harvard Pilgrim Health Care Institutional Review Board approval to conduct the study. We imported them into StataSE V.11.2 (StataCorp, College Station, Texas). All analyses used the Survey commands of Stata to account for the clustered sample design and to adjust for the disproportioned allocation of sample clusters between the capital (or most densely populated) region and other areas of a country. In combined analyses, we weighted each country survey population equally rather than proportional to country population size in order to prevent multi-country results from being dominated by the Nigeria results. At the time of data collection, the WHO/MeTA surveys did not collect information on malaria diagnostic tests. In the absence of confirmed diagnosis with a parasite-based test, clinical suspicion of malaria and presumptive treatment are based on the presence of fever [17,18]. Therefore we included in our analysis all individuals with acute symptoms recorded under the group of symptoms “fever, headache, hot body”.

We hypothesized that episodes of acute fever would be treated differently in the public and private sectors because changes in national antimalarial policies at the time of the surveys targeted primarily public healthcare facilities. We used the Stata survey multivariate logistic regression commands to identify predictors of the decision to seek care in a public healthcare facility rather than in the private sector and of subsequent use of antimalarials and ACT in presence of acute fever.

Potential predictor variables included in the model were health services-related (sources of care accessed during acute fever), household-related (location, socio-economic status, proximity of healthcare facilities, respondent education, respondent opinions on medicines access) and patient-related (patient gender, age under 5 years old, associated symptoms, perceived severity of fever). Socio-economic satus of households was assessed from the self-reported monthly expenditure quintile; expenditure quintile boundaries were calculated from the most recent national household economic survey adjusted for household size. Households in the lowest quintile were considered poor; households in the other quintiles were considered non-poor.

## Results

### Characteristics of surveyed households

The characteristics of all 5261 surveyed households, including prevalence of acute illness, have been previously described [15]. Table 1 presents the differences observed between the households surveyed in capitals and in other parts of the countries, and between poor and non-poor households. In capital areas, a lower proportion of households was poor (estimated proportion: 10.8% vs. 35.6%, p < 0.001), situated far from public healthcare facilities (9.1% vs. 15.3%, p < 0.05), and far from private healthcare providers (16.3% vs. 35.0%, p < 0.001) or medicines retail outlets (7.5% vs.15.3%, p < 0.05). Higher proportions of respondents living in capital areas were female (66.5% vs. 48.3%, p < 0.001), between 25–50 years old (76.9% vs. 66.5%, p < 0.001), with education beyond high school (26.3% vs.18.3%, p < 0.05), and of the opinion that medicines are affordable (53.4% vs. 42.2%, p < 0.05). Poor households shared many of the attributes of households outside capital areas. In addition, the proportion of households stocking medicines at home was lower if households were poor (39.4% vs. 52.6%, p < 0.001), even though a higher proportion of poor respondents reported an acute illness in the preceding 2 weeks (55.4% vs. 48.7%, p = 0.01).

**Table 1:** **Demographic and socio-economic characteristics of surveyed households**

**Households**	Poor*	Non-poor*		Outside capital**	Within capital**	
	**1485**	**3676**		**4188**	**973**	
**Mean household size (95% Confidence Intervals)**	**7.0(6.5,7.5)**	**6.3(5.9,6.8)**		**6.5(6.1, 6.8)**	**6.8(5.5, 8.0)**	
	**Proportion Estimates**			**Proportion Estimates**		
	**(95% Confidence Intervals)**			**(95% Confidence Intervals)**		
			**Pearson chi2**			**Pearson chi2**
Outside Capital	**90.6(87.8, 93.4)**	**67.8(63.3, 72.3)**	**p<0.001**	1.00(-,-)	0.0(0.0, 0.0)	na
Poor	1.00(-,-)	0.0(0.0, 0.0)	na	**35.6(31.2, 40.1)**	10.8(7.5, 14.1)	**p<0.001**
With children under 15 years old	**91.2(89.2, 93.1)**	**85.4(83.3, 87.6)**	**p<0.001**	88.1(86.3, 89.9)	84.2(80.1, 88.4)	ns
With children 5 years old	**65.6(62.2, 68.9)**	**59.3(55.8, 62.7)**	**p<0.001**	60.5(57.6, 63.4)	62.8(56.0, 69.5)	ns
Respondent						
Female	49.3(44.5, 54.1)	54.5(51.2, 57.7)	ns	**48.3(44.7, 51.9)**	**66.5(61.7, 71.4)**	**p<0.001**
Age 25-50	**64.7(61.3, 68.2)**	**71.0(68.5, 73.4)**	**p<0.01**	66.5(64.3, 68.7)	76.9(71.8, 82.0)	**p<0.001**
Complete at least primary school	**43.7(38.5, 48.9)**	**63.5(58.7, 68.2)**	**p<0.001**	57.0(52.7, 61.4)	59.5(48.7, 70.2)	ns
Education high school or above	**7.2(5.0, 9.5)**	**25.8(22.3, 29.2)**	**p<0.001**	18.3(15.3, 21.3)	26.3(19.4, 33.3)	**p<0.05**
Opinions						
Closet public facility has medicines	40.9(35.6, 46.2)	36.3(32.1, 40.4)	ns	38.9(34.8, 43.0)	33.8(25.9, 41.7)	ns
Free medicines at public facility	**61.4(54.9, 68.0)**	**50.7(45.5, 56.0)**	**p<0.001**	56.0(50.1, 62.0)	47.5(38.1,56.9)	ns
Can usually afford medicines needed	**35.8(30.9, 40.7)**	**48.9(44.8, 53.0)**	**p<0.001**	**42.2(38.3,46.0)**	**53.4(44.0M,62.8)**	**P<0.05**
**Distance from closet public health care facility**						
Less than 15 mins	49.2(42.2, 55.8)	49.6(45.5,53.7)	ns	51.1(46.1,56.0)	44.8(37.1,52.4)	ns
Over 1 hour	**19.3(14.1, 24.4)**	**11.4(8.8,13.9)**	**p<0.005**	**15.3(11.9,18.7)**	**9.1(4.8,13.3)**	**p<0.05**
**Distance from closet private health care provider**						
Less than 15 mminutes	**31.1(25.0, 37.1)**	**40.7(35.5,45.9)**	**p<0.005**	**34.2(29.3,39.1)**	**48.7(36.6,60.8)**	**p=0.01**
Over 1 hour	**41.8(34.9,48.7)**	**25.5(21.0,29.9)**	**p<0.001**	**35.0(29.8,40.2)**	**16.3(7.0,25.6)**	**p<0.001**
**Distance from closet private medication retail outlet**						
Less tahn 15 minutes	62.1(55.3,68.8)	67.9(63.3,72.6)	ns	65.9(60.5,71.4)	67.0(58.4,75.6)	ns
Over 1 hour	**18.8(13.4,24.2)**	**11.0(7.7,14.4)**	**p<0.01**	**15.3(11.4, 19.1)**	**7.5(1.2,13.8)**	**p<0.05**
**With medicines at home**	**39.4(35.1,43.6)**	**52.6(49.4,55.9)**	**p<0.001**	47.6(44.2,51.0)	52.1(46.2,57.9)	ns
**With anitimarial at home**	9.1(7.2,11.0)	10.8(9.2,12.3)	ns	10.6(9.0,12.1)	9.4(6.9,11.8)	ns
**Reporting Illness(es) in past two weeks**	**55.4(50.4,60.3)**	**48.7(45.2,52..1)**	**p=0.01**	50.5(46.6,54.4)	50.9(44.6,57.3)	ns
**Reporting fever epidode(s) in past two weeks**	37.2(32.9,41.5)	33.7(30.4,36.9)	ns	34.1(30.8,37.4)	36.5(29.9,43.1)	ns

*Respondent self-seclection into lowest pre-define country-specific expenditure based on data from recent national household surveys reporting per capita Gross National Income or Consumption.

**The WHO survey methodology calls for surveying the “capital or most densely populated area in the country. The capital region was surveyed in Ghana, Kenya and Uganda; in Nigeria, logos was selected as the most densely populated area of the country.

Of the 2000 individuals with a recent febrile episode, 91.8% sought care outside the home, 96.0% took medicines, 67.0% received antimalarials, and 16.2% received ACT. Several characteristics differed between sick individuals from households in capital vs. non-capital areas. In capital areas, lower proportions of respondents reported a severe fever (12.6% vs. 21.8%, p < 0.001), were treated in a public healthcare facility (43.6% vs. 53.6%, p < 0.05), received ACT (9.0% vs. 18.5%, p < 0.05) or antibiotics (20.7% vs. 34.8%, p < 0.005). (Table 2) The proportion of sick individuals from poor households who sought care outside the home was lower (88.3% vs. 93.4, p < 0.005), the proportion traveling more than one hour to receive care was higher (23.3% vs. 13.1%, p < 0.001), and the proportion receiving analgesics or NSAIDS was lower (65.2% vs. 70.6%, p < 0.05) than in the group from non-poor households.

**Table 2:** **Demographic characteristics, severity and actions taken for episodes of acute fever in the two weeks preceding the survey according to socio-economic status and geographic location**

**Individuals with recent acute fever (N)**	**Poor**	**Non-Poor**		**Outside Capital**	**Within Capital**	
	**652**	**1384**		**1671**	**329**	
	**Proportion Estimates(95% Confindence Intervals)**		**Pearson chi2**	**Proportion Estimates(95% Confindence Intervals)**		**Pearson chi2**
**Country**			**p<0.001**			**p<0.001**
Gambia	8.2(5.1,13.2)	20.2(12.8,30.4)		6.8(4.3,10.8)	25.9	
Ghana	9.0(5.1,15.3)	14.7(10.0,21.0)		15.2(10.1,22.3)	5.6(1.8,16.2)	
Kenya	33.9(23.5,46.1)	18.3(12.3,26.4)		29.2(20.7,39.5)	4.3(1.6,11.3)	
Negeria	16.4(9.7,26.4)	20..4(14.2,28.4)		21.2(14.5,29.8)	12.7(5.0,28.9)	
Uganda	32.5(22.1,44.9)	26.4(18.3,36.1)		27.6(19.3,37.9)	30.5(13.7, 54.9)	
**Female**	52.7(48.3,57.2)	55.9(52.3,59.3)	ns	54.1(51.2,57.0)	57.4(50.0,64.4)	ns
**Age umder**	44.4(38.8,50.1)	36.5(33.2,40.0)	ns	40.3(36.7,44.1)	34.7(229.9,39.9)	ns
**Very severe fever**	19.6(15.8,24.1)	19.6(17.1,22.4)	ns	**21.8(19.1,24.9)**	**12.6(9.5,16.4)**	**p<0.001**
**Reported associated symptoms**			ns			ns
Upper respiratory (cough, runny nose, sore throat, earache)	33.5(27.9,39.6)	36.0(31.4,40.8)	ns	36.8(32.4,41.4)	30.1(22.7,38.7)	ns
Difficulty breathing, fast breathing	7.1(5.1,9.8)	6.0(4.5,7.9)	ns	7.1(5.6,9.1)	3.8(2.0,7.3)	ns
Gastro-interstinal (diarrhea, nausea, could not eat)	25.1(20.8,29.9)	22.8(19.0,27.0)	ns	23.5(20.5,26.8)	23.5(15.1,34.7)	ns
All other symptoms	35.9(28.44.2)	42.2(37.2,47.3)	ns	38.7(33.0,44.8)	44.8(35.5,54.4)	ns
**Actions taken**						
Sought care outside home	**88.3(84.4,91.4)**	**93.4(91.8,94.8)**	**p<0.005**	91.1(88.8,92.9)	94.3(91.6,96.1)	ns
traveled more than one hour to recieve care	**23.3(16.6,31.6)**	**13.1(10.6,15.9)**	**p<0.001**	17.4(13.7,22.0)	12.5(8.3,18.5)	ns
received in a public health facility	52.1(46.1,58.0)	50.8(46.1,55.4)	ns	**53.6(49.2,57.9)**	**43.6(33.7,54.0)**	**p<0.05**
**Treatment**						
Received medicines	95.3(92.6,97.0)	96.4(94.8,97.5)	ns	96.6(95.3,97.5)	94.4(89.5,97.0)	ns
obtained all medicines free-of-charge	31.2(25.4,37.7)	30.6(26.1,35.6)	ns	32.8(27.7,38.8)	24.4(17.7,32.6)	ns
Received antimalarials	64.2(59.1,68.9)	68.3(64.9,71.5)	ns	67.5(63.9,70.9)	65.4(59.6,70.8)	ns
obtained antimalarials and other medicines in public facility	36.7(31.1,42.7)	33.5(29.8,37.4)	ns	36.7(40.9)	27.5(20.2,36.2)	ns
received ACT	18.5(13.9,24.2)	15.2(12.2,18.7)	ns	**18.5(15.2,22.4)**	**9.0(4.7,16.7)**	**p<0.05**
obtained ACT and other medicines in public in facility	13.5(9.8,18.5)	10.6(8.3,13.5)	ns	**14.4(11.5,17.8)**	**2.7(1.1,6.2)**	**p<0.0001**
obtained all medicines including ACT free-of-charge	10.3(7.0,15.0)	6.9(5.0,9.4)	ns	**9.9(7.4,13.1)**	**1.7(0.6,4.5)**	**p<0.001**
Received antibiotics	31.3(26.9,36.0)	31.4(27.5,35.6)	ns	**34.8(31.2,38.5)**	**20.7(14.28.5)**	**p<0.005**
Received analgesics or NSAIDs	**65.2(59.9,70.2)**	**70.6(67.3,73.8)**	**p<0.05**	69.3(66.2,72.3)	67.6(58.7,75.4)	ns

### Use of antimalarials and ACTs to treat acute fever

**Figure 1:**
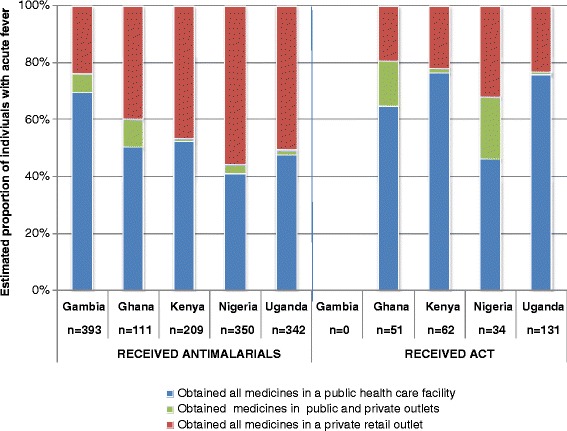
**Sources of antimalarials in the community.**

The profile of antimalarials used to treat acute fever varied greatly across surveys, and the proportion of fevers treated with ACT varied from none in Gambia to 26.6% in Ghana. In the Gambia survey, chloroquine, SP, and quinine were the only antimalarials reported to treat fever, while a variety of different antimalarial medications were documented in the four other surveys. (Table 3) When ACT was used, the choice of artemisinin combination usually appeared to be consistent with national policies in effect at the time of the surveys: i.e., artesunate + amodiaquine (AS + AQ) in Ghana, artemether + lumefantrine in Kenya, Nigeria, and Uganda. The proportion of individuals with acute fever who obtained all the medicines prescribed or recommended in a public healthcare facility was consistently higher in the group of patients who received ACT (Figure 1).

**Table 3:** **Use of antimalarials among individuals with acute fever and treated with antimalarials (proportion estimates & 95% confidence intervals)**

	**Gambia**	**Ghana**	**Kenya**	**Nigeria**	**Uganda**
Artemisinin-combination theraphy					
artemether_amiodaquine	-	-	-	0%(0%, 1%)	-
artemether_lumefantrine	-	4%(-1%)	30%(21%,39%)	8%(4%,11%)	36%(26%,46%)
artesunate_amodiaquine	-	32%(21%,42%)	-	0%(0%,1%)	-
artesunate_mefloquine	-	-	0%(0%,1%)	-	-
dihydroartemisinin_piperaquine	-	5%(1%,9%)	0%(0%,1%)	-	1%(0%,2%)
**Other Antimalarials**					
SP	64%(54%,74%)	4%(0%,7%)	13%(6%,20%)	9%(3%,15%)	9%(6%,13%)
chloroquine	97%(95%,100%)	8%(1%,16%)	1%(0%,2%)	39%(29%,48%)	18%(12%,24%)
amodiaquine	-	21%(13%,29%)	13%(8%,18%)	0%(0%,1%)	4%(1%,7%)
antimalarial, unspecified	-	19%(11%,27%)	33%(25%,42%)	38%(27%,48%)	24%(13%,35%)
quinine	1%(0%,1%)	4%(-1%,8%)	11%(6%,15%)	1%(0%,3%)	21%(17%,25%)
artemether	-	3%(0%,5%)	-	3%(0%,6%)	1%(0%,1%)
artemisinin	-	-	1%(0%,2%)	1%(0%,2%)	-
artesunate	-	9%(4%,14%)	-	5%(2%,8%)	-
chloroquine_SP	1%(0%,2%)	-	-	-	0%(0%,1%)
dihydroartemisinin	-	-	2%(0%,3%)	1%(0%,1%)	-
halofantrine	-	-	-	1%(0%,2%)	-
mefloquine	-	-	-	0%(0%,1%)	-
quinine_SP	-	-	2%(0%,3%)	-	-

### Predictors of seeking care in a public healthcare facility

Having hypothesized that the point of care would be a key determinant for receiving ACT, we examined which factors led people to seek care in a public healthcare facility. The strongest determinant was proximity: compared to people living within 15 min of a public health facility, people were less likely to seek care in the public sector if their home was located between 15 minutes and 1 hour away (odds ratio 0.40; 95% confidence intervals (0.29–0.56), P < 0.001] and even less likely when travel time was over one hour away [0.21, (0.14–0.33), P < 0.001]. (Table 4) The further away people lived from a private medicines retail outlet, the more likely they were to seek care in a public healthcare facility: from [1.20, (0.83–1.75), not significant (NS)] when travel time to a private outlet was between 15 minutes and one hour to [1.57, (1.06–2.33), P < 0.05] when travel time increased to over one hour.

**Table 4:** **Predictors of seeking care in a public healthcare facility, of receiving an antimalarial, and of receiving ACT**

	**Sought care in a Public Health care facility**	**Received an antimalarial**	**Received ACT**
**Number of observations**	**1686**	**1686**	**1257**
	**OR(95% CI)**	**OR(95% CI)**	**OR(95% CI)**
**Household-related predictors**			
**Sought care in a public health care facility**	**-**	1.67***	4.64***(2.98,7.22)
**Non-capital region (vs capital region)**	2.14***(1.36,3.37)	1.52*(1.00,2.30)	0.93(0.40,2.15)
**Total monthly expenditure in lowest quintile (vs higher)**	0.92(0.66,1.30)	0.80(0.59,1.09)	1.04(0.72,1.49)
**Distance from public hospital or health facility (vs <15min)**			
15 minutes to 1 hour	0.40***(0.29,0.56)	0.95(0.68,1.33)	0.79(0.52,1.21)
Over 1 hour	0.21***(0.14,0.33)	0.65*(0.43,0.98)	0.72(0.37,1.40)
**Distance from private hospital, clinic, or doctor (vs <15min)**			
15 minutes to 1 hour	1.49*(1.02,2.16)	1.14(0.82,1.60)	1.02(0.63,1.66)
Over 1 hour	2.75***(1.91,3.98)	1.13(0.79,1.62)	1.15(0.65,2.02)
**Distance from private medicines retail outlet (vs <15min)**			
15 minutes to 1 hour	1.20(0.83,1.75)	1.16(0.80,1.67)	1.42(0.84,2.41)
Over 1 hour	1.57**(1.06,2.33)	0.80(0.53,1.20)	1.56(0.89,2.71)
**Respondent education high school or above (vs less or none)**	0.91(0.64,1.28)	1.17(0.79,1.74)	2.67***(1.74,4.11)
**Respondent opinions (vs no or unsure)**			
Closet public facility usually has medicines	1.47*(1.09,1.99)	1.25(0.92,1.71)	1.03(0.69,1.54)
free medicines at public facility	1.91***(1.45,2.51)	0.80(0.59,1.07)	1.20(0.77,1.88)
Can usually medicines needed	0.65**(0.48,0.89)	1.20(0.87,1.66)	0.96(0.66,1.40)
**Patients -related predictors**			
Female (vs male)	0.90(0.71,1.13)	0.90(0.70,1.16)	0.87(0.65,1.17)
Age (vs 15 and over)			
Under 5 years	1.34(0.99,1.80)	1.28(0.91,1.79)	0.80(0.57,1.13)
5-15 years	1.07(0.76,1.51)	1.21(0.80,1.83)	1.07(0.70,1.64)
**Associate symptoms (vs no)**			
URI (cough, runny nose, sore throat, earache)	0.95(0.72,1.25)	0.59***(0.44,0.79)	0.68*(0.48,0.99)
Diarrhea, vomiting, nausea, could not eat	0.99(0.73,1.35)	1.40*(1.01,1.94)	1.40(0.97,2.02)
Difficulty breathing, fast breathing	1.41(0.85,2.37)	0.88(0.56,1.40)	0.76(0.43,1.35)
Other symptomes	0.73*(0.54,0.98)	0.61***(0.46,0.82)	0.99(0.65,1.51)
Perceived illness severity (vs mild)			
Moderate	1.36*(1.02,1.82)	1.82***(1.38,2.40)	1.32(0.82,2.10)
Severe	1.46*(1.01,2.11)	1.39(0.9,2.12)	0.83(0.48,1.44)

*p<0.05, **p< 0.01, ***p<0.001.

While people were also more likely to seek care in a public healthcare facility if they lived in non-capital regions (2.14, (1.36–3.37), P < 0.01), education and poverty did not appear to directly affect their choice of source of care. Other factors that increased the likelihood of seeking care in a public healthcare facility were the perceptions that the closest public healthcare facility usually carries the medicines needed [1.47, (1.09–1.99), P < 0.05] and that it is possible to obtain free medicines in public healthcare facilities [1.91, (1.45–2.51), P < 0.001]. Respondents who felt that they could afford medicines were less likely to visit a public facility [0.65, (0.48–0.89), P < 0.01]. Finally, the likelihood of visiting a public facility increased with the perceived severity of illness [from 1.36, (1.02–1.82), P < 0.05 for illness perceived as moderately severe to 1.46, (1.01–2.11), P < 0.05 for illness perceived as very severe].

### Predictors of antimalarial and ACT use for acute fever

Seeking care in a public health facility increased the likelihood of receiving an antimalarial for a febrile episode [1.67, (1.24–2.25), P < 0.01], as did living outside of a country’s capital area [1.52, (1.00–2.30), P < 0.05]. (Table 4) In addition, the perceived severity of illness and presence of gastro-intestinal symptoms increased the odds of receiving antimalarials [1.82, (1.38–2.40), P < 0.001 and 1.40, (1.01–1.94), P < 0.05 respectively], while upper respiratory infection (URI) symptoms and other less frequent symptoms decreased the odds [0.59, (0.44–0.79), P < 0.001 and 0.61, (0.46–0.82), P < 0.001 respectively].

By far, the most significant determinant of receiving ACT was being treated in a public healthcare facility [4.64, (2.98–7.22), P < 0.001]. Respondent education beyond high school was also associated with ACT use [2.67, (1.74–4.11), P < 0.001]. The presence of associated URI symptoms decreased the odds of receiving ACT [0.68, (0.48–0.99), P < 0.05]. No other significant associations were observed between ACT treatment and the potential predictors tested in the model.

## Discussion

Overall, our results underscore the high prevalence of febrile episodes responsible for high health services utilization and antimalarials use in both adults and children. A third of households reported at least one recent episode of fever. Over 90% of individuals with acute fever received treatment outside the home and reported using medicines, two-third of them received antimalarials. Published reports suggest a decrease in the true prevalence of malaria in Sub-Saharan Africa at the time of the surveys, well before the dissemination of rapid diagnostic testing (RDT) [19,20]. Yet, the use of antimalarials was very high in all five surveys irrespective of geographic location, socioeconomic status or age. Our results document widespread presumptive antimalarial treatment for febrile symptoms, a practice responsible for antimalarial overuse and emergence of antimalarial resistance. They highlight the importance of restricting antimalarials to diagnosed cases of malaria with the use of RDTs, as recommended by current WHO guidelines [21].

A key finding was the poor compliance of African communities with national policies recommending ACT as first line treatment for uncomplicated episodes of malaria. When the surveys took place in 2007–2008, national ACT policies had been implemented for at least three years in four of the five countries, and yet we found that ACT was used in only 16% of the reported febrile episodes. Our results document the gap between pharmaceutical policies in place and the reality on the ground. The highest observed ACT use was in Ghana where only 32% of individuals taking antimalarials received AS-AQ, which had been first-line treatment of malaria for four years. In addition, several instances of inappropriate artemisinin monotherapy were reported in the four countries where ACT was available and the use of halofantrine was documented in the Nigeria survey despite its ban one year earlier [12].

Results from recent *ACTwatch* surveys suggest that the majority of children under 5 years old with acute fever are treated at home or in the private sector [22]. However, our analysis of the WHO household surveys indicates that more than half of individuals complaining of fever seek care in public healthcare facilities. This difference may be related to the different sampling methodologies. The WHO surveys seek to draw a broader picture of community treatment decisions about seeking care and obtaining medicines. They apply no restrictive criteria to the random selection of households, collecting information on all household members, all their recent symptoms, and all medicines taken and kept at home. *ACTwatch* surveys strictly focus on children under 5 years old, excluding households without febrile children under 5 years old. In the five surveys we analyzed, only 39% of individuals with fever were children under 5 years old. In addition, the *ACTwatch* instrument attempts to capture the iterative steps that caregivers go through from the first recognition of fever in a child, while the WHO survey examines medicines use during the entire episode of illness [23].

Our study indicates that half of febrile episodes were seen in public healthcare facilities. This highlights the critical role played by the public sector in treating common ailments in communities, evidence that needs to be taken into account when designing programs to strengthen healthcare systems and eradicate malaria. Moreover, health professionals in the public sector were more likely than other healthcare providers to prescribe antimalarials and ACT; seeking care in a public healthcare facility not only increased the odds or receiving an antimalarial but was the most important determinant of receiving ACT at the time of the surveys, a result concordant with other recent reports [12,24]. Of concern, children under 5 years old, who are more vulnerable to severe disease and most in need of proper antimalarial treatment, had a slightly lower likelihood of receiving ACT than adults even though their likelihood of visiting a public healthcare facility and receiving antimalarials was somewhat higher. This finding could be related to the lack of appropriate ACT pediatric formulations at the time of the surveys. It advocates for systematically evaluating the effects of antimalarial policies in the entire community, in order to identify unintended consequences that may occur in key subgroups.

Our results illustrate how much perceptions about the health system shape healthcare behaviors of households, in particular opinions about where medicines can be best accessed, where they are most affordable, and which providers are best suited to treat more severe symptoms. As expected, convenience also played a role: households were more likely to seek care in a public facility when it was close by and when they lived in less densely populated areas where public facilities often are the only available source of care. Notably, we did not find an association between living in less densely populated regions and access to ACT, despite the higher use of public health facilities in these areas. A possible explanation is that public facilities in capital regions may have had a better supply of ACT at the time of the surveys, although this cannot be determined from our data.

The strong relationship between education and ACT use is consistent with evidence that people with higher education exhibit better recall of antimalarial names [25]. The presence of associated URI symptoms may increase the likelihood of receiving antibiotics at the expenses of antimalarials, as previously reported [15], which would explain the negative association between URI symptoms and any type of antimalarial treatment.

Finally, our results indicate that national ACT policies did not discriminate against the poor at the time of the surveys when program implementation was largely confined to the public sector. This goes against early concerns about ACT not reaching the poor; however, this situation may have changed since then and evidence of inequity is emerging with the recent development of private channels for ACT access [13,26].

The standardized WHO methodology to measure access to and use of medicines in communities of low- and middle-income countries with rapid sample surveys has several limitations in design, including underrepresentation of hard to reach areas, as well as potential variance underestimation, misclassification of households in socio-economic brackets, and underestimation of self-medication [15]. In addition, the survey depends on the respondents’ identification of medicines used; about a third of antimalarials were reported as “antimalarial,” which may have resulted in an underestimation of ACT use. While the WHO surveys did not have a special focus on malaria, results are consistent with other surveys focusing on ACT coverage [27-29]. They also provide valuable information about determinants of care seeking and patterns of antimalarial use in the early years of ACT national policy implementation. Such surveys can be recommended as affordable strategies to assess the community impact of future national policy changes to eradicate malaria, such as malaria immunization campaigns [30].

## Conclusion

Access to ACT remained limited in five African countries in the early years of national policies encouraging its use as first-line treatment for malaria. It was largely confined to public sector health facilities where many households sought more affordable care. Since households continue to use antimalarials to treat most febrile episodes, there is a critical need for expanded use of RDT to guide appropriate treatment by community providers and caregivers.
